# Electrocardiogram in Endomyocardial Fibrosis

**Published:** 2011-10-02

**Authors:** Jaganmohan A Tharakan

**Affiliations:** Professor of Cardiology, Sree Chitra Tirunal Institute for Medical Sciences and Technology, Trivandrum, Kerala

**Keywords:** endomyocardial fibrosis, electrocardiogram

Endomyocardial fibrosis (EMF) is a distinct obliterative cardiomyopathy characterized anatomically by right/left/bi ventricular endocardial fibrotic thickening and varying degrees of obliteration of the ventricular cavity. Chamber obliteration leads to diastolic restriction to ventricular filling and thus diastolic ventricular dysfunction.  Involvement of the chordae tendinae and papillary muscles in the fibrotic process results in atrioventricular (AV) valve regurgitation. The ventricular endocardial thickening is predominantly at apex leading to apical obliteration. When it extends to the inflow, papillary muscles and chordae are involved and AV valve regurgitation ensues. The hemodynamics is that of a restrictive cardiomyopathy with varying degrees of AV valve regurgitation.

Though Arthur Williams and Davis [1,2] described LV fibrosis with mitral valve incompetence as early as 1938 and 1948 respectively in Uganda, the first autopsy report of EMF in India by Samuel and Anklesaria [3] in Kerala by Gopi came as late as in 1960 and 1962 respectively. The diagnosis was clinical and hence the patients were diagnosed late in the course of their disease. Even though electrocardiaogram (ECG) was available, the lack of sensitivity and specificity of ECG in diagnosis as well as prognostication relegated its role as a useful clinical tool. However with the advent of hemodynamic and angiographic diagnostic facilities, the disease was more reliably and accurately diagnosed and the disease spectrum better characterized, including grades of cavity obliteration and AV valve regurgitation of the right ventricular (RV), left ventricular (LV) or both [4,5]. Availability of echocardiography made  non-invasive diagnosis and hemodynamic assessment of EMF easy, and  an occasional patient was detected to have asymptomatic EMF or EMF associated with other acquired heart diseases. Magnetic resonance cardiac imaging further refined appreciation of fibrotic endocardial involvement in EMF.

ECG abnormalities or ECG changes paralleling the extent of obliteration and AV valve regurgitation and resulting  hemodynamic derangement has not been systematically studied. We will discuss briefly the structural abnormality and the consequent hemodynamic abnormality, and the associated ECG abnormalities of EMF.

RV EMF results in RV diastolic dysfunction and tricuspid valve incompetence. It also results in a distorted and often contracted RV cavity and RA enlargement due to RV diastolic dysfunction and or tricuspid valve incompetence.

Moderate to large pericardial effusion is common in severe forms of RV EMF with severe tricuspid incompetence and heart failure. The ECG often suggests right atrial (RA) abnormality,  reflecting  RA pressure and volume overload . ECG abnormality includes peaking and increased P wave amplitude in lead II and rarely tall peaked and narrow positive P waves resembling the Himalayan P waves of Ebstein's anomaly. Often we have observed  QR pattern with a diminutive R wave in lead V1 (Figure 1),  attributed to RA enlargement, though this pattern is seen even in patients with atrial fibrillation (AF)  (Figure 2). In a recent study of ECG in isolated RV EMF, we noted  a dominant R wave in V2 in the absence of QR pattern in V1 in 14 of 25 patients resulting in early transition from a dominant S wave in right sided chest leads  to dominant R wave in V2 or earlier  (Figure 3). Atrial arrhythmias are common and AF is the end stage atrial rhythm in most patients with advanced RV EMF. It is noteworthy that patients with RV EMF and AF rarely have fast ventricular response, in striking contrast to patients with AF and LV EMF. Patients with large pericardial effusion often have low voltage QRS, satisfying the low voltage ECG criteria.

LV EMF is characterized by varying degrees of endocardial obliteration generally limited to the endocardium and only secondary effects on myocardium and no pericardial involvement. Morphologically, LV hypertrophy is uncommon and the cavity dilates in the transverse axis to accommodate the increased preload resulting from mitral incompetence. The fibrotic areas can calcify and endocardial calcification with a contracted LV cavity is highly specific for EMF. The electrocardiogram reflects the hemodynamic abnormality. Left atrial (LA) abnormality in ECG parallels the diastolic dysfunction and degree of mitral incompetence. AF occurs in advanced and uncorrected case of LVEMF. The odd finding is a uniform ST segment depression and T wave inversion more evident in the lateral chest leads, similar to apical hypertrophic cardiomyopathy (HCM) and non- ST elevation acute coronary syndromes (Figure 4). The ECG and echocardiographic abnormalities of severe LV EMF and Apical HCM closely resemble one another. Endocardial calcification, plastering of posterior mitral leaflet to posterior LV endocardium  and AV valve incompetence favour LV EMF. Not uncommonly, nonspecific ST T wave abnormalities in the precordial leads in a routine ECG evaluation brings these patients to clinical attention for further evaluation to exclude coronary heart disease and LVEMF is diagnosed during  echocardiographic study or LV angiogram. Patients with LVEMF and resultant pulmonary hypertension leads to have features of RV hypertrophy and right ward frontal plane QRS axis deviation. Ventricular tachycardia and sudden death attributable to malignant ventricular arrhythmia is rare.

More than 50% patients with EMF have biventricular involvement and the ECG reflects  a combination of these abnormalities. ECG findings reported  by Balakrishnan et al [6] from Sree Chitra Tirunal Institute for Medical Sciences and Technology, Trivandrum  (210 patients) in 1991 included AF (33%), junctional rhythm or heart block (4.4%), right axis deviation (29%), intra ventricular conduction abnormality (16%) and atrial tachycardia or atrial flutter(3.5%)6. Earlier three large studies of 60 patients, 95 patients and 50 patients, reported AF in 40%, junctional rhythm in 2%, RA enlargement in 32% and QR pattern in V1 in 25% patients [7-9]. LA enlargement was common in patients with LVEMF and 18% of LVEMF patients had LVH (left ventricular hypertrophy)  with strain [9].

Electrophysiological abnormalities in EMF have not been widely studied. In 1990, we conducted electrophysiological studies in 18 patients with EMF who were in sinus rhythm. The important findings included, lower AV Wenkebach heart rate on incremental atrial pacing, mild increase in AH interval and a normal HV interval except in one patient (HV interval 70 ms). Interestingly, the atria had pockets of low voltage splintered atrial activity (electrical myopathy of RA as a fore runner to atrial arrhythmias and AF). The injury potentials obtained from the RV endocardium had low voltages corresponding to areas of fibrotic  endocardial involvement. The pacing threshold at the fibrotic areas  was surprisingly within normal range [10].

Incidence of complete heart block (CHB) or sick sinus syndrome (SSS) necessitating pacing is rare in EMF. We had 3 patients with RV EMF and CHB requiring pacing. The two patients seen in the Eighties, had epicardial pacing after failure to obtain stable pacing with tined lead in the RV due to lack of trabeculae. Subsequently, endocardial active fixation lead was used  in the third patient with acceptable pacing threshold. SSS patients are offered dual chamber or VVI pacing using active fixation leads. In  RVEMF patients,  AF does not result in a rapid ventricular response unlike in LVEMF. Patients subjected to surgery presently also undergo RA reduction surgery to facilitate maintenance of sinus rhythm.

We have observed a significant reduction in patients with a new diagnosis of EMF over the past 2 decades [11]. The newly diagnosed patients are older, less symptomatic, have less severe form of the disease, are often diagnosed when evaluated for non specific symptoms and ST T wave abnormality in ECG, and have excellent medium term prognosis (<10% mortality  in 7 years). ECG is neither specific nor sensitive for the diagnosis of EMF but  in the last decade, many patients have been diagnosed as LVEMF  during investigation for non specific ST T changes in ECG.

EMF remains an enigma for want of an etiopathogenic explanation. The incidence appears to be decreasing and end stage EMF is rarely seen now. Less affected patients are often diagnosed when evaluated for non specific symptoms and an abnormal  ECG. ECG is neither sensitive nor specific to rule in or rule out EMF and meticulous echocardiographic examination is investigation of choice to detect milder forms of EMF.

## Figures and Tables

**Figure 1:**
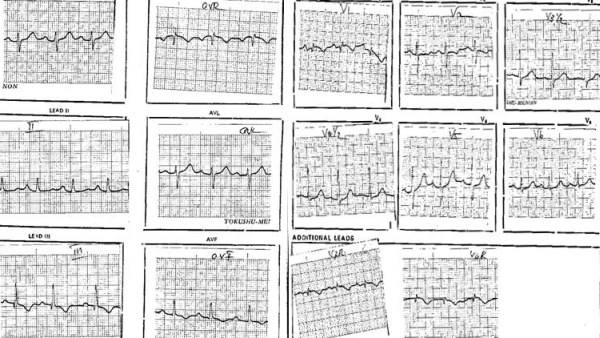
Patient with RVEMF: QR pattern in V1, peaked P wave in lead II

**Figure 2:**
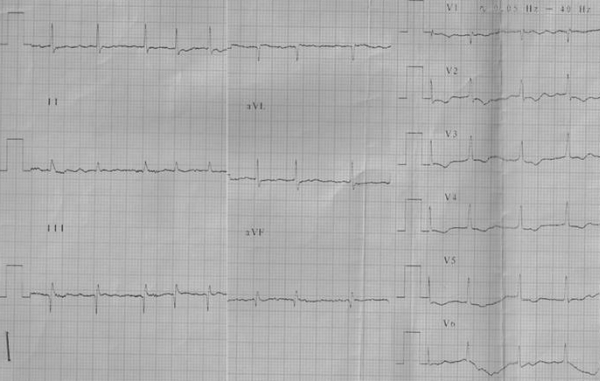
Patient with RV EMF: AF and qR in V1

**Figure 3:**
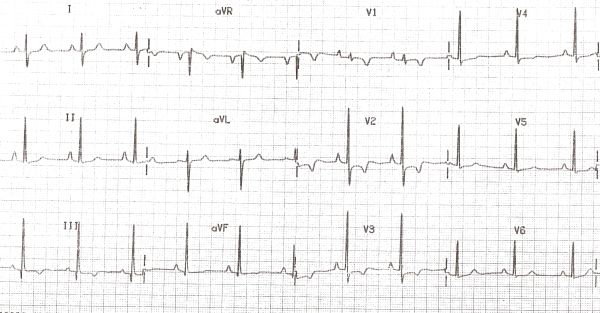
Patient with RVEMF: Early transition of QRS at V2

**Figure 4:**
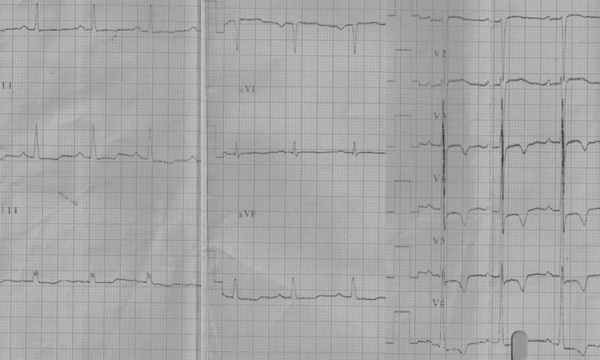
Patient with LV EMF: LVH with strain  and LAE

## References

[R1] Davies JNP (1948). Endocardial fibrosis in Africans. East African Medical Journal.

[R2] Davies JNP (1955). The pathology of endomyocardial fibrosis in Uganda. Br. Heart Journal.

[R3] Samuel I (1960). Endomyocardial fibrosis in South India. Ind J Path Bact.

[R4] Sasidharan K, Sapru RP (1983). Radiological features of Endomyocardial fibrosis. Endomyocardial fibrosis in India.

[R5] Tharakakan JM, Valiathan MS (1993). Angiographic features of Endomyocardial fibrosis. Endomyocardial fibrosis.

[R6] Balakrishnan KG, Valiathan MS (1993). Clinical course of patients in Kerala. Endomyocardial fibrosis.

[R7] Sapru RP, Sapru RP (1983). Clinical profile of endomyocardial fibrosis. Endomyocardial fibrosis in India.

[R8] Vijayaraghavan G, Sapru RP (1983). Endomyocardial fibrosis: Clinical, ECG and radiological features. Endomyocardial fibrosis in India.

[R9] Jacob G, Sapru RP (1983). Endomyocardial fibrosis in Kerala. Endomyocardial fibrosis in India.

[R10] Bhat A (1985). Electrophysiological abnormality in EMF.

[R11] Tharakan J (2009). Current perspective on endomyocardial fibrosis. Current  Science.

